# Auditory risk recognition is socially transmitted across territory borders in wild birds

**DOI:** 10.1007/s10071-024-01858-6

**Published:** 2024-03-02

**Authors:** Jakub Szymkowiak

**Affiliations:** 1 Faculty of Biology, Forest Biology Center, Adam Mickiewicz University, Uniwersytetu Poznańskiego 6, 61-614 Poznan, Poland; 2 Population Ecology Research Unit, Faculty of Biology, Institute of Environmental Biology, Adam Mickiewicz University, Uniwersytetu Poznańskiego 6, 61-614 Poznan, Poland

**Keywords:** Anti-predator behavior, Cultural transmission, Eavesdropping, Information networks, Social learning

## Abstract

Prey species commonly assess predation risk based on acoustic signals, such as predator vocalizations or heterospecific alarm calls. The resulting risk-sensitive decision-making affects not only the behavior and life-history of individual prey, but also has far-reaching ecological consequences for population, community, and ecosystem dynamics. Although auditory risk recognition is ubiquitous in animals, it remains unclear how individuals gain the ability to recognize specific sounds as cues of a threat. Here, it has been shown that free-living birds (Wood Warblers *Phylloscopus sibilatrix*) can learn to recognize unfamiliar, complex sounds (samples of punk rock songs) as cues of a threat from conspecifics holding adjacent territories during the spring breeding season. In a playback experiment, Wood Warblers initially ignored the unfamiliar sounds, but after repeatedly hearing that these sounds trigger alarm calling reaction of neighbors, most individuals showed an anti-predator response to them. Moreover, once learned soon after nestlings hatching, the anti-predator response of parents toward previously unfamiliar sounds was then retained over the entire nestlings rearing period. These results demonstrate that social learning via the association of unfamiliar sounds with known alarm signals enables the spread of anti-predator behavior across territory borders and provides a mechanism explaining the widespread abilities of animals to assess predation risk based on acoustic cues.

## Introduction

Predation is a key selective pressure in nature. To counter the risk of predation, prey species have evolved a range of mechanisms allowing them to assess that risk and flexibly adjust the behavior to the perceived threat (Lima and Dill 1990; Caro 2005). Risk recognition occurs via various sensory modalities, including reliance on acoustic signals such as vocalizations produced by predators or alarm calls of heterospecifics with shared enemies (Caro 2005; Magrath et al. 2005; Hettena et al. 2014; Carlson and Griesser 2022). Auditory risk recognition is common in animals and affects prey’s behavior, physiology, and life history traits, which further scale-up to affect prey’s survival and demography (Caro 2005; Zanette et al. 2011; LaManna and Martin 2016; Suraci et al. 2016; Dudeck et al. 2017; Allen et al. 2022). In turn, this may have far-reaching ecological consequences for population, community, and ecosystem dynamics, e.g., via behaviorally mediated trophic cascades that emerge from prey’s threat-sensitive decision-making and permeate down through multiple levels of a food web (Suraci et al. 2016). However, there is an unresolved puzzle regarding the phenomenon of auditory risk recognition in wild animals: how prey species recognize that specific sounds convey information about a threat?

In animals, risk recognition is usually not ontogenetically fixed, but involves constant learning about what is risky and what is not (Griffin 2004; Caro 2005; Crane and Ferrari 2013; Rowell et al. 2020). Learning allows prey to acquire novel anti-predator responses and adjust the pre-existing ones; thus, it is a key aspect of an adaptive anti-predator behavior. In birds, for example, individuals can learn to recognize novel alarm signals by associating these signals with the appearance of a predator (Magrath et al. 2015). Theory predicts, however, that when learning through personal experience is costly, such as during predator–prey interactions, individuals should learn socially, that is, by observing others (Galef and Laland 2005; Hoppit and Laland 2013; Potvin et al. 2018). Social learning may enable the transmission of anti-predator responses toward predators and indirect cues of predation risk (e.g., predator odor) from knowledgeable individuals to naïve ones, ultimately expanding the repertoire of anti-predator behaviors of the latter at lower cost than asocial learning. Learning from others to recognize predators and indirect cues of predation risk appears taxonomically widespread (Griffin 2004; Crane and Ferrari 2013; Rowell et al. 2020) and could, therefore, provide a mechanism through which individuals learn auditory risk recognition. However, current examples of social learning about predator risk are limited mainly to studies in captivity and naïve individuals learning to recognize threat visually or based on olfactory cues (reviewed in Griffin 2004; Caro 2005; Crane and Ferrari 2013; Rowell et al. 2020). In contrast, social learning of auditory risk recognition under natural conditions received much less research attention (Potvin et al. 2018; Dutour et al. 2019; Szymkowiak 2021), even though risk assessment via acoustic signals by prey is ubiquitous in the wild and social learning appears as a key mechanism of non-genetic acquisition of an anti-predator behavior in animals.

Here, it has been tested experimentally whether an ability to recognize threat via auditory cues can be socially transmitted among birds occupying adjacent territories during the spring breeding season. Specifically, it was tested whether Wood Warblers (*Phylloscopus sibilatrix*)—small passerines inhabiting temperate European forests—can learn to recognize unfamiliar sounds as cues of a threat, by associating the appearance of these sounds with alarm calling reaction of conspecific neighbors.

Auditory risk recognition is common in birds (Eggers et al. 2006; Emmering and Schmidt 2011; Zanette et al. 2011; Hettena et al. 2014; Dudeck et al. 2017; Szymkowiak and Thomson 2019; Allen et al. 2022) and recent evidence suggest that social learning via the association of unfamiliar sounds with known alarm signals may provide a plausible mechanism of its transmission in avian communities (Potvin et al. 2018; Dutour et al. 2019; Szymkowiak 2021). In particular, Superb-fairy Wrens (*Malurus cyaneus*) were recently shown to learn to recognize novel heterospecific alarm calls by associating them with the already known alarm calls (Potvin et al. 2018). Similarly, Great Tits (*Parus major*) were found to learn to recognize previously unfamiliar sounds as alarm signals by associating the unfamiliar sounds with conspecific mobbing calls (Dutour et al. 2019), and this form of learning can also occur between species (Szymkowiak 2021). Thus, it is likely that learning via acoustic–acoustic association allows social transmission of anti-predator behavior among territorial birds. During the spring breeding season, most bird species in the temperate zone occupy distinct territories, where nests are placed, and offspring is raised. When a threat to the offspring appears close to the nest, parents usually suspend nestlings’ feeding and produce alarm calls, which are audible and publicly available (Caro 2005). These calls alert other individuals breeding within an auditory distance about a danger (Szymkowiak 2022), but could also facilitate learning about novel risk cues among territorial neighbors, possibly resulting in naïve individuals acquiring anti-predator response toward previously unfamiliar sounds if these repeatedly prompt alarm calling in conspecifics or heterospecifics (Curio et al. 1978; Griffin 2004; Crane and Ferrari 2013; Potvin et al. 2018; Rowell et al. 2020; Szymkowiak 2021).

## Methods

### Study population and area

**Fig. 1:**
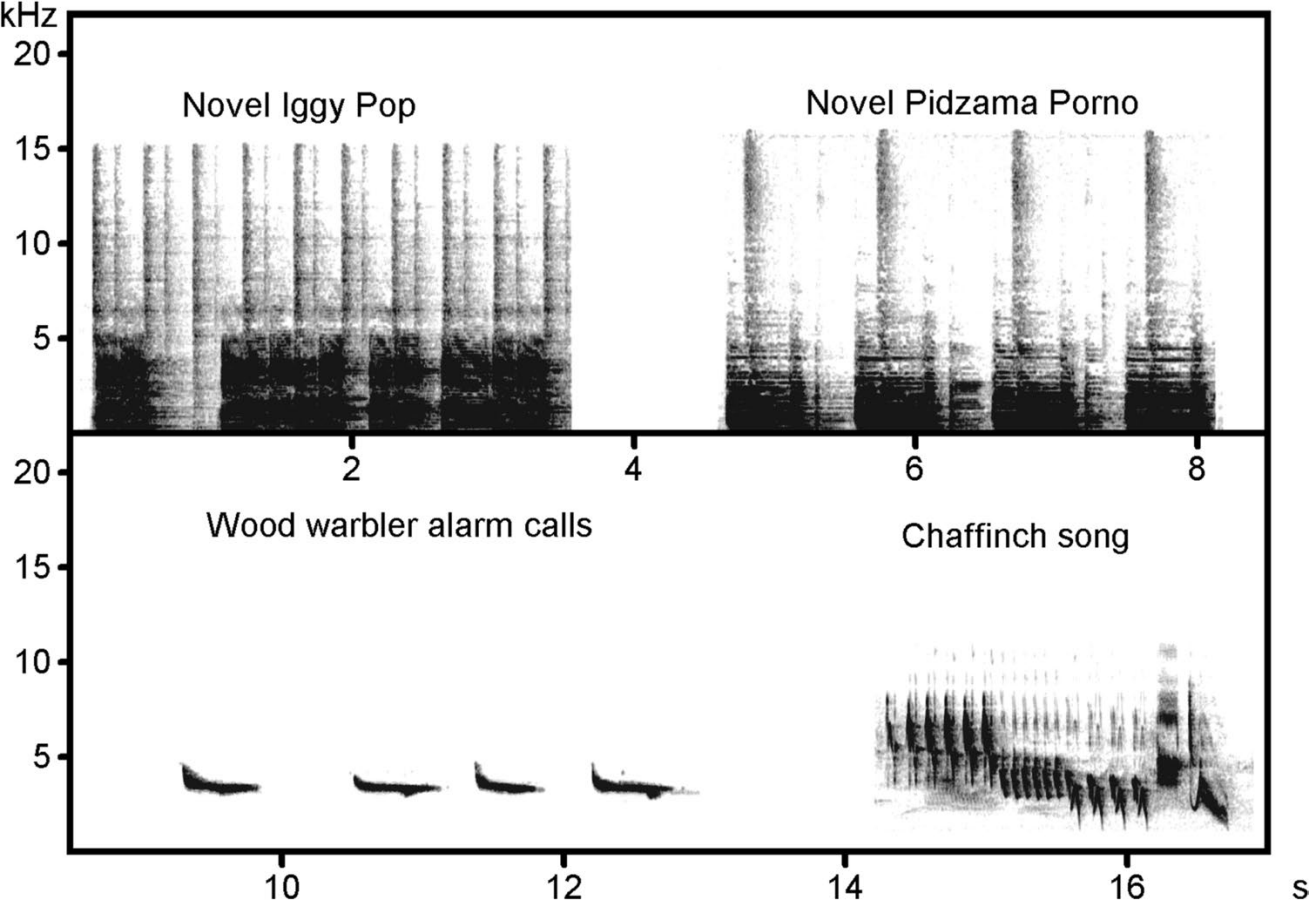
Sonograms of sounds used in the social learning experiment. Sounds were visualized in Avisoft-SASLab Pro (window: Blackman, FFT length: 1024, frame size: 75%, overlap: 75%)

The study was conducted in a wild population of Wood Warblers breeding in the Wielkopolska National Park, western Poland (52°16ʹ N, 16°47ʹ E). Wood Warblers are small (c. 10 g), territorial songbirds breeding in temperate European forests and wintering in equatorial Africa. They build dome-shaped nests on the forest floor, in which altricial nestlings (typically 5–6) are born and raised by both parents and ca. 10-day post-hatching, they are capable to leave the nest (Wesołowski 1985). Undisturbed parents feed nestlings silently, but suspend feeding of young and produce simple, uniform alarm calls when a threat appears close to the nest (Fig. 1; Maziarz et al. 2018). These calls are accessible to conspecific neighbors, as individuals during the spring breeding season usually occupy territories within the active space of alarm signals of multiple conspecifics (Szymkowiak 2022).

The experiment was conducted in 2020, during the spring breeding season, on two large plots (c. 138 ha and 60 ha, respectively; spaced 3.5 km apart) consisting of continuous forest habitat. As part of the long-term study on Wood Warbler’s ecology and behavior, all territorial individuals were censused throughout the breeding season (from mid-April to early-July) on both study plots using the territory mapping method (Szymkowiak et al. 2016, 2017; Szymkowiak and Thomson 2019). All nests found were georeferenced and monitored.

### Social learning experiment

The experiment consisted of four stages: pre-training, training, and two after-training tests. In the pre-training tests, the behavioral response (alarm calling and feeding visit latency) of 12 Wood Warbler pairs to playbacks of 2 novel sounds broadcasted close to their nests was assessed to ensure that they initially ignored both sounds. To exclude any innate or earlier-learned responses, artificial sounds, that is, ~ 3.5 s sample intro riffs from two punk rock songs were used (*The Passenger* by Iggy Pop and *Outsider* by Pidżama Porno, novel IP and novel PP, respectively). As a control sound, the song of a Chaffinch (*Fringilla coelebs*), a widespread songbird in temperate forests, recorded at the study area was used. Parents were tested at the beginning of offspring rearing period, when their nestlings were 1–2 days old. The playbacks were broadcasted when both parents were simultaneously close to the nest and feeding young undisturbed, using iPod touch 32 GB connected via Bluetooth with a JBL Flip 4 loudspeaker placed on a tree ~ 10 m from the nest of a focal pair. Nests of focal pairs were spaced at least 200 m apart to avoid inadvertent learning between study subjects later during the experiment (c.f. Szymkowiak 2022). At this stage of the experiment, parents are expected to respond to both novel sounds in a similar strength as to playbacks of Chaffinch songs.

Training playbacks mimicked alarm calling reaction of conspecifics to the appearance of a novel sound. The birds were trained to novel sounds by playing back one of the two novel sounds (either IP or PP, hereafter training sound) along with simultaneous broadcasting of conspecific alarm calls representing natural responses of parents to the presence of a high threat predator close to the nest (see *Playback stimuli* for details). Each focal pair received 5 training playbacks over 1 day, with the first training beginning 45–120 min after the last pre-training playback. Five pairs were trained to novel sound IP and seven pairs were trained to the novel sound PP. In addition, focal pairs were exposed to five playbacks of the other novel sound but without simultaneous broadcasting of alarm calls (hereafter non-training control). This later allowed testing for sensitization, i.e., whether the mere repeated exposure to playbacks of a novel sound could itself lead to increased response to this sound (Shettleworth 2010). During training, playbacks of novel sounds and alarm calls were broadcasted using a set of two speakers placed on trees ~ 10 m apart each other and ~ 50 m from the nest of a focal pair, which falls between the minimum (40 m) and Q_25%_ (65 m) nearest-neighbor distance among Wood Warbler territories at the study site (Szymkowiak 2022). One speaker broadcasted training sound, while the other speaker broadcasted a bout of alarm calls, thereby simulated conspecific tutors nesting away. Alarm call playbacks started 2 s after the beginning of the training sound playback, which resulted in a training sound appearing together with a chorus of conspecific alarm calls and resembled a natural scenario in which calling reaction of parents is retained for some time after threat disappearance (Maziarz et al. 2018). Playbacks of non-training control sounds were broadcasted between consecutive playbacks of training sounds, using one of the two speakers. Position of loudspeakers between consecutive training playbacks was altered by ~ 5 m, although keeping constant the general direction from which a focal pair heard the playbacks.

Post-training tests assessed whether focal pairs learned to recognize training sounds as cues of a threat by testing again their anti-predator responses (alarm calling and feeding visit latency) to training and non-training control sounds. The first post-training test was conducted a day after the last training session. The second post-training test was conducted 7–9 days later, close to nestlings fledging time, to assess whether parents retained the learned response (if any) over the entire nestlings rearing period. At this stage of the experiment, social learning hypothesis predicts stronger anti-predator response of Wood Warblers to training sounds than to non-training control sounds, which should be apparent in both post-training tests if the learned response was retained. On the contrary, increased response to both sounds would imply simple sensitization following repeated playbacks. One nest was depredated before the first post-training test and further four before the second post-training test; thus, eleven and seven pairs were tested during the first and second post-training test, respectively.

### Playback stimuli

Playbacks of novel sounds were prepared based on ~ 3.5 s sample intro riffs from *The Passenger* song by Iggy Pop (album: *Lust for Life*, 1977) and *Outsider* song by Pidżama Porno (album: *Ulice jak stygmaty–absolutne rarytasy*, 1999) (Fig. 1). Playback stimuli for pre- and post-training tests were the same and included three samples of either sound IP or PP interspersed by 3–5 s of silence. For training playbacks, five unique stimuli (one per training session) of each novel sound were prepared, each based on the same ~ 3.5 s sample of a novel sound but with different intervals of silence (3–5 s).

Control playback stimuli for pre-training tests included three songs of a Chaffinch (each 2.5 s long, interspersed by 4–6 s of silence) recorded at the study area with Sennheiser ME67/K6 directional microphone and Zoom H6 handy recorder (Fig. 1). The recordings were filtered using fast Fourier transform (FFT) filter with 1 and 10 kHz threshold (high and low pass, respectively) to remove background noise.

Training playbacks of alarm calls lasted for 45–60 s (median = 51 s) and included bouts of 48–66 calls (median = 55 calls). Bouts of alarm calls prompted using the taxidermy mount of Eurasian Jay (*Garrulus glandarius*) were used; thus, the calls represented natural responses of parents to the presence of a high threat predator close to the nest (Maziarz et al. 2018). Alarm calls were recorded in the local population, using Sennheiser ME67/K6 directional microphone connected to Zoom H6 handy recorder. Prior playback preparation, the background noise in recordings was removed using the lasso selection tool and then reducing the amplitude to minimal values. In total, five unique playback stimuli (one per training session) were prepared, each based on alarm calls recorded from a different pair.

All playbacks were prepared in Adobe Audition CS6 as 16-bit wav files (sampling rate = 44.1 kHz) and adjusted their amplitude to match 80 dB(A) SPL at 1 m from the loudspeaker (measured using CEM DT-8852 sound level meter).

### Behavioral scoring

Bird responses to pre- and post-training playbacks were recorded using Nikon d7200 DSLR camera with Nikkor 18–105 mm lens and Sennheiser ME67/K6 shotgun microphone, camouflaged with a masking net and placed 10–15 m away from the nest of a focal pair. A typical anti-predator response of parents during nestlings rearing is alarm calling and the cessation of nestlings feeding (Maziarz et al. 2018; Szymkowiak 2022). Thus, based on field recordings, the total number of alarm calls produced by a pair and the latency to first feeding visit by a parent (1 s accuracy) for the 3 min after playback started were calculated. In case no feeding visit was observed for 3 min following the start of a trial, feeding visit latency was scored 181 s. Behavioral scoring based on field recordings was blind with respect to the stage of the experiment and playback role.

### Statistical analyses

The number of alarm calls and the latency to first feeding visit measured in response to playbacks were non-independent and correlated (*r* = 0.62). Thus, both behavioral responses were combined into a single principal component (PC1, explaining 81.2% of variance), which was then used in subsequent analyses as a compound measure of the strength of parents’ anti-predator response to playbacks.

Linear mixed models (LMMs) were used to compare Wood Warbler responses (PC1) to playbacks. To avoid model overparameterization, separate models were built for pre- and post-training comparisons. All models included pair ID as random effects, to account for multiple testing of the same breeding pairs. When comparing pre-training responses, the LMM included playback (novel IP vs. novel PP vs. Chaffinch song) as explanatory term, as well as the number and age of nestlings as covariates, as these may affect parents’ anti-predator behavior at the nest (Caro 2005; Królikowska et al. 2016). When comparing post-training responses, sound role during training (training vs. non-training control), time of post-training test (post-training test 1 or 2), and interaction between both variables were included as explanatory terms. The covariates were nestlings’ number and age, as well as the type of a sound (IP vs. PP). The social learning hypothesis predicts stronger response of Wood Warblers to training sounds than to non-training control sounds, and the lack of interaction between playback role (training vs. non-training) and time of post-training test (post-training test 1 or 2) if the learned response was retained over the two post-training tests. All analyses were conducted in R ver. 3.4.1 (R Core Team 2018) using packages *nFactors* ver. 2.3.3 (Raiche 2010) and *glmmTMB* ver. 1.0.2.1 (Brooks et al. 2017).

## Results

**Fig. 2:**
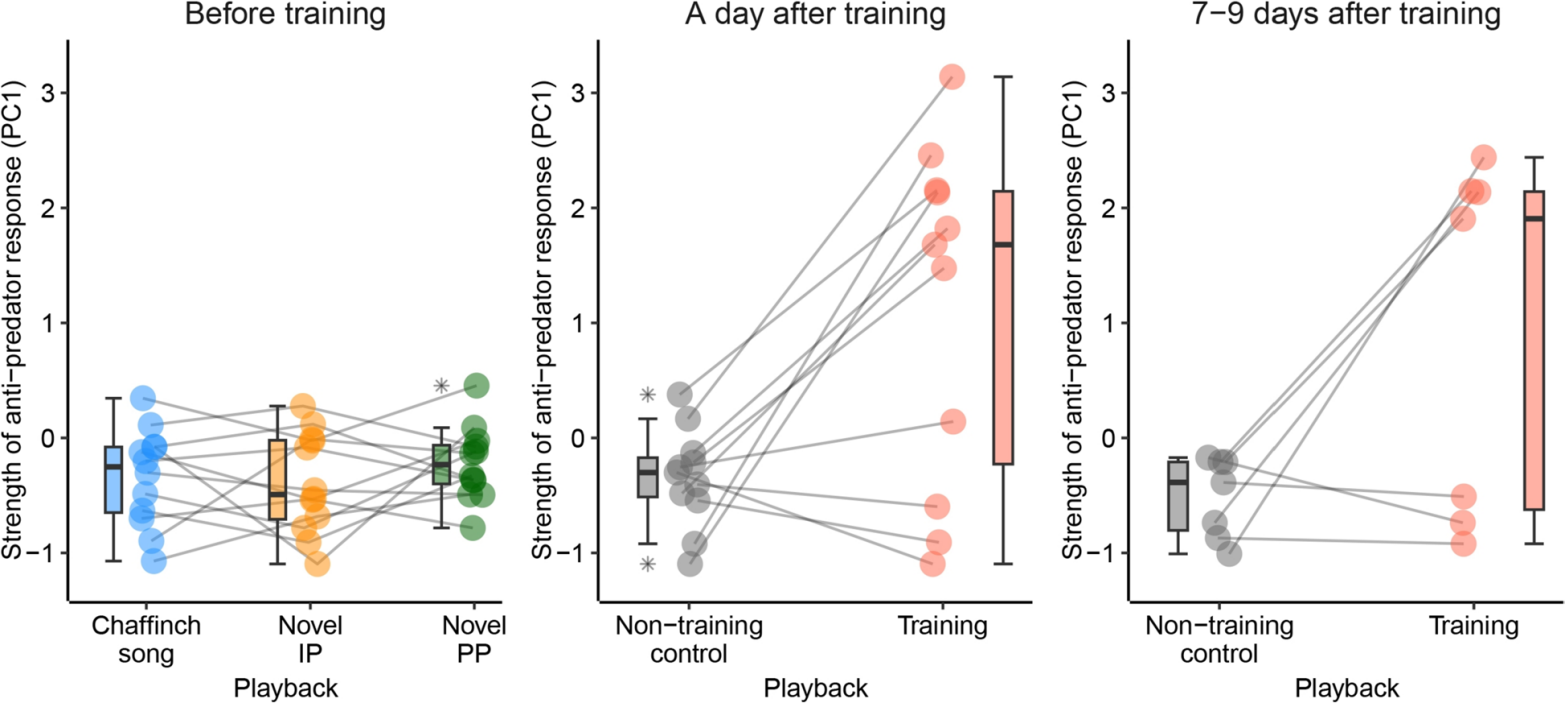
Before training, focal pairs responded to novel sounds (*The Passenger* by Iggy Pop and *Outsider* by Pidżama Porno, novel IP and novel PP, respectively) as to the neutral Chaffinch song. Y-axis shows scores of the first principal component, with higher values representing stronger anti-predator response, i.e., more alarm calls and higher latency to first feeding visit after playback. A day after training, most pairs responded stronger to training sounds than to the non-training control sounds. Stronger anti-predator response to training sounds was also observed during the second post-training test conducted 7–9 days after training

Before training, Wood Warblers responded to both novel sounds as to the neutral Chaffinch song (playback effect: Wald’s *χ*^2^ = 1.82, df = 2, *P* = 0.402; Fig. 2). Bird responses to playbacks did not depend on the number (Wald’s *χ*^2^ = 0.09, df = 1, *P* = 0.766) and age of nestlings (Wald’s *χ*^2^ = 0.18, df = 1, *P* = 0.672). After training, bird responses to playbacks of novel sounds depended on sound role during training, with focal pairs showing stronger anti-predator response to training sounds (i.e., producing alarm calls and suspending nestlings feeding), as compared to the non-training control sounds (sound role effect: Wald’s *χ*^2^ = 27.86, df = 1, *P* < 0.001; Fig. 2). Importantly, response strength did not depend on the time of post-training test and was similar at the beginning and the end of nestlings rearing period (sound role × time of post-training test effect: Wald’s *χ*^2^ = 0.02, df = 1, *P* = 0.884; Fig. 2).

Specifically, stronger responses to training than control playbacks during the first post-training test were observed in 7 pairs (out of 11 tested, Fig. 2). Due to nest depredation, responses of seven pairs were tested during the second post-training test, including four pairs that responded to training sounds in the first post-training test. These four pairs also responded stronger to training playbacks during the second post-training test, while the other three pairs that ignored both novel sounds in the first pre-training test consistently ignored them also in the second post-training test (Fig. 2). Neither the number (Wald’s *χ*^2^ = 0.19, df = 1, *P* = 0.661) nor the age of nestlings (Wald’s *χ*^2^ = 0.003, df = 1, *P* = 0.956) had an effect on post-training responses. Jointly, these results demonstrate that most focal pairs learned socially to recognize the specific training sounds as cues of a threat and once learned soon after nestlings hatching, the anti-predator response of parents was then retained until the end of nestlings rearing period.

## Discussion

Animals routinely rely on acoustic signals when assessing predation risk, but the role of social learning in the acquisition of such skills under natural conditions remain poorly explored. Here, it has been shown that after short training in an ecologically relevant scenario, most Wood Warblers responded to initially unfamiliar sounds by suspending nestlings feeding and producing alarm calls. This is a typical anti-predator response of parents to the appearance of a threat in the vicinity of a nest (Maziarz et al. 2018). These results demonstrate that under natural conditions, anti-predator responses toward previously unfamiliar sounds can be socially transmitted among territorial individuals, with naïve birds learning through the association of unfamiliar sounds and alarm calling reactions of conspecific neighbors. Moreover, it was found that once learned soon after nestlings hatching, the anti-predator response of parents was then retained until the end of nestlings rearing period. To be adaptive, the learned anti-predator response has to be memorized and retained by individuals for later use, but—except for primates—earlier studies examined the persistence of socially learned anti-predator responses mainly over short time-frames after the behavior was learned (reviewed in Crane and Ferrari 2013). Here, it has been shown that at the beginning of a pivotal phase of a breeding cycle, birds can acquire a vital life skill—recognition of novel risk cues—from conspecific neighbors, which they can incorporate into their own repertoire of anti-predator behaviors and use later when taking care of own nestlings. Jointly, these results demonstrate social learning as one of the mechanisms explaining the widespread abilities of animals to assess predation risk via acoustic signals.

The present study design does not allow to unequivocally determine whether focal pairs perceived treatment sounds as vocalizations of a novel predator or alarm calls of a novel heterospecific. Indeed, recent work has shown that the recognition of novel heterospecific alarm calls can be learned via this mechanism (Potvin et al. 2018; Dutour et al. 2019; Szymkowiak 2021). Here, however, birds learned to recognize artificial sounds with more complex acoustic structure than usually relatively simply structured alarm calls, which points toward the efficiency and flexibility of learning to recognize novel auditory risk cues via acoustic–acoustic association. Thus, under natural conditions, learning by associating unfamiliar sounds with already known alarm signals is likely to operate in both contexts and enable individuals to learn to recognize both novel heterospecific alarm calls and novel predator vocalizations.

Learning via acoustic–acoustic association is likely a common mechanism of social transmission of auditory risk recognition in birds. During the spring breeding season, most bird species in the temperate zone are bound to distinct territories. Yet, acoustic signals allow information exchange and learning even when individuals are spaced apart each other and does not require naïve individuals to see alarm calling tutors or a novel predator/novel heterospecific itself (Potvin et al. 2018; Dutour et al. 2017; Szymkowiak 2021; this study). In a natural scenario, this likely allows naïve individuals to acquire predator-awareness at lower cost, without risking the potentially fatal direct interaction with a predator (Griffin 2004; Galef and Laland 2005; Crane and Ferrari 2013; Hoppit and Laland 2013). Thus, social transmission of anti-predator behavior via acoustic association is likely to be especially common in populations of territorial birds. In Wood Warblers, for example, individuals during the spring breeding season are intertwined in population-wide information webs, with most of them occupying territories within the active space of alarm signals of multiple conspecifics (Szymkowiak 2022). Thus, even though individuals are territorial and spaced apart each other, information can readily spread throughout the population via alarm calls and facilitate learning about novel risk cues. Moreover, in multi-species communities, gaining risk-related information and learning will be further facilitated by eavesdropping on alarm calls of heterospecifics with shared enemies (Randler and Vollmer 2013; Wheatcroft and Price 2013; Magrath et al. 2015; Dutour et al. 2017, 2019; Potvin et al. 2018; Szymkowiak 2021; Carlson and Griesser 2022). Association of the already recognized alarm signals and unfamiliar sounds is, therefore, likely an important mechanism of social learning in territorial birds (and possibly also other sound-oriented animals), enabling rapid acquisition of novel risk cues recognition and its efficient spread across species and territory borders.

Social transmission of risk recognition among territorial conspecifics can have important eco-evolutionary consequences. Traditionally, co-occurring conspecifics have been considered as putative competitors and the negative impacts of competitive interactions on individuals’ fitness and higher order ecological phenomena (e.g., negative density-dependent population regulation) have been emphasized, particularly in case of non-grouping, territorial animals (MacArthur and Levins 1964; Fretwell and Lucas 1969; Begon et al. 2005). However, the results of this study point toward the positive role of individuals’ social environment in the acquisition of vital, fitness-enhancing skills (predator risk recognition) even in otherwise territorial animals. Apart from being competitors, conspecifics are also the repositories of information, which they can transfer to other individuals (Holt 2007; Szymkowiak and Schmidt 2022). In turn, this may result in adaptive changes in the behavior of initially naïve individuals due to the enhanced abilities to recognize threat (Griffin 2004; Crane and Ferrari 2013; Rowell et al. 2020). Consequently, social transmission of anti-predator behavior has the potential to alter the overall outcome of competitive interactions by directly affecting the strength and direction of intraspecific density dependence, as well as social selection pressures among interacting individuals in a way that promotes coexistence (Seppänen et al. 2007; Gil et al. 2017, 2018, 2019).

Unraveling what factors determine whether individuals learn auditory risk recognition via acoustic–acoustic association will be an important next step for further understanding how, under natural conditions, this behavior is acquired by individuals and then socially transmitted throughout populations. Here, most birds learned to recognize previously unfamiliar sounds as cues of a threat, but ~ 35% did not show any response to treatment sounds. There are several possible explanations for the lack of response including, for example, habituation to repeated playbacks (Rowell et al. 2020) or differences in learning phenotypes of individuals being trained (Aplin 2016). For instance, learning about risk usually requires very few exposures to a predator or an indirect predator cue, as it would be maladaptive to require many experiences with a predator before learning to recognize it as a threat (Rowell et al. 2020). Nevertheless, inter-individual differences in learning performance may result in some individuals requiring more trials to learn. Moreover, risk responsiveness may also depend on factors not controlled here, e.g., personality traits, with bold individuals being more risk prone than shy individuals exhibiting more risk-averse strategy (Cole and Quinn 2014). One limitation of the present study is a relatively small sample size, stemming from the experiment being conducted under natural conditions, on free-living birds. While the learned responses to treatment sounds were clear and consistent across experimental stages, the present study does not allow exploring factors affecting inter-individual differences in learning performance. As learning by associating familiar and unknown acoustic signals appears to be an important mechanism of socially acquired auditory risk recognition in birds (Potvin et al. 2018; Dutour et al. 2017; Szymkowiak 2021; this study), examining specifically how various factors affect learning at the individual level and what factors determine its social transmission at the population level appears to be a fruitful avenue for future research.

In conclusion, it was found that under natural conditions, an ability to recognize novel acoustic risk cues can be socially transmitted among birds occupying adjacent territories during the spring breeding season. Once learned, the anti-predator response to novel risk cues is then retained by individuals for later use. Jointly, these results demonstrate social learning as being at the forefront of predator–prey interactions and providing one of the mechanisms underlying the widespread abilities of animals to rely on acoustic signals when assessing predation risk.

## Data Availability

All data generated or analyzed during this study are available from a corresponding author upon any request.
